# Polygenic burden associated to oligodendrocyte precursor cells and radial glia influences the hippocampal volume changes induced by aerobic exercise in schizophrenia patients

**DOI:** 10.1038/s41398-019-0618-z

**Published:** 2019-11-11

**Authors:** Sergi Papiol, Daniel Keeser, Alkomiet Hasan, Thomas Schneider-Axmann, Florian Raabe, Franziska Degenhardt, Moritz J. Rossner, Heike Bickeböller, Ludovico Cantuti-Castelvetri, Mikael Simons, Thomas Wobrock, Andrea Schmitt, Berend Malchow, Peter Falkai

**Affiliations:** 1 Department of Psychiatry, University Hospital, Nussbaumstrasse 7, 80336 Munich, Germany; 2 Institute of Psychiatric Phenomics and Genomics (IPPG), University Hospital, Ludwig Maximilian University, Nussbaumstrasse 7, 80336 Munich, Germany; 3 Institute of Clinical Radiology, Ludwig Maximilian University Munich, Marchioninistrasse 15, 81377 Munich, Germany; 4 International Max Planck Research School for Translational Psychiatry, Kraepelinstr. 2-10, 80804 Munich, Germany; 5 Institute of Human Genetics, University of Bonn, Bonn, Germany; 6 Department of Genetic Epidemiology, University Medical Center, Georg-August-Universität Göttingen, Humboldtallee 32, 37073 Göttingen, Germany; 7 German Center for Neurodegenerative Diseases (DZNE), Feodor-Lynen Str. 17, 81377 Munich, Germany; 8 Munich Cluster for Systems Neurology (SyNergy), 81377 Munich, Germany; 9 Institute of Neuronal Cell Biology, Technical University Munich, 80805 Munich, Germany; 10 Department of Psychiatry and Psychotherapy, County Hospitals Darmstadt-Dieburg, Krankenhausstrasse 7, 64823 Groß-Umstadt, Germany; 11 Laboratory of Neuroscience (LIM27), Institute of Psychiatry, University of Sao Paulo, Rua Dr. Ovídio Pires de Campos 785, Sao Paulo-SP, 05403-903 Brazil

**Keywords:** Schizophrenia, Personalized medicine

## Abstract

Hippocampal volume decrease is a structural hallmark of schizophrenia (SCZ), and convergent evidence from postmortem and imaging studies suggests that it may be explained by changes in the cytoarchitecture of the cornu ammonis 4 (CA4) and dentate gyrus (DG) subfields. Increasing evidence indicates that aerobic exercise increases hippocampal volume in CA subfields and improves cognition in SCZ patients. Previous studies showed that the effects of exercise on the hippocampus might be connected to the polygenic burden of SCZ risk variants. However, little is known about cell type-specific genetic contributions to these structural changes. In this secondary analysis, we evaluated the modulatory role of cell type-specific SCZ polygenic risk scores (PRS) on volume changes in the CA1, CA2/3, and CA4/DG subfields over time. We studied 20 multi-episode SCZ patients and 23 healthy controls who performed aerobic exercise, and 21 multi-episode SCZ patients allocated to a control intervention (table soccer) for 3 months. Magnetic resonance imaging-based assessments were performed with FreeSurfer at baseline and after 3 months. The analyses showed that the polygenic burden associated with oligodendrocyte precursor cells (OPC) and radial glia (RG) significantly influenced the volume changes between baseline and 3 months in the CA4/DG subfield in SCZ patients performing aerobic exercise. A higher OPC- or RG-associated genetic risk burden was associated with a less pronounced volume increase or even a decrease in CA4/DG during the exercise intervention. We hypothesize that SCZ cell type-specific polygenic risk modulates the aerobic exercise-induced neuroplastic processes in the hippocampus.

## Introduction

Hippocampal volume decrease has been consistently reported in first- and multi-episode schizophrenia (SCZ) (e.g., refs. ^1–4^). This structural change has been associated with psychopathological severity and cognitive deficits in SCZ patients^5–11^. Despite the relevance of both negative and cognitive symptoms for disability and functional recovery^12^, to date pharmacological strategies have shown little success in the management of these symptoms^13–15^.

An increasing body of evidence indicates that aerobic exercise as an add-on strategy may help to improve these disability-related symptoms in SCZ patients^16–18^. Studies in the general population reported the beneficial effects of physical activity on cognitive performance and brain structure and function^19–21^, and two recent meta-analyses of these studies provided remarkable evidence that aerobic exercise, including moderate-intensity continuous training, increases hippocampal volumes^22,23^. These results suggest that neuroplastic processes in this brain region could drive symptom improvement in SCZ patients.

The first study to investigate the effects of aerobic exercise on brain structure in SCZ was performed in a small sample of multi-episode patients and reported an increase in hippocampal volume after 3 months of aerobic endurance training^24^. Subsequent studies with similar designs could not replicate these structural findings^16–18^, however, although they did observe beneficial effects on patients’ global functioning^17^ and training-related volume increases of the left superior, middle, and inferior anterior temporal gyri^16^. The type, intensity, and duration of the exercise intervention may explain the differences in the results of the aforementioned studies, at least in part^25–27^. Recently, a study with a design that closely resembled the methodology of the above-mentioned first study in SCZ^24^ replicated the positive effects of aerobic exercise on hippocampal volume in chronic SCZ patients^28^.

Imaging studies that used automatic methods for hippocampus subfield segmentation recently revealed that cornu ammonis (CA) regions CA1–4 and the dentate gyrus (DG) show more volume reduction than other hippocampal regions in both first-episode and chronic SCZ; the reductions are larger in the left hemisphere and correlated to cognitive deficits^29–32^. Postmortem studies from our group confirmed these volumetric changes in the anterior and posterior hippocampus and provided clues to understand the cytoarchitecture of these changes. We observed a decreased number of oligodendrocytes in the left and right CA4 (posterior hippocampus) and left CA4 (anterior hippocampus) and fewer neurons in the left DG^33,34^. Moreover, the number of oligodendrocytes correlated with the volume of CA4^34^, and the reduction in the number of oligodendrocytes in the left CA4 was more pronounced in patients with definitive cognitive deficits^35^. It is unknown, however, whether the number of oligodendrocyte precursor cells (OPCs) or mature oligodendrocytes is reduced. Impaired differentiation of OPCs has been hypothesized in SCZ^36^ and represents an interesting field for functional studies with patient-derived induced pluripotent stem cells^37^. Radial glia (RG) cells are the common progenitors of neurons and oligodendrocytes, and during development they give rise to neurons and glia cells^38^. In the hippocampus and cortex of adult mouse brains, they retain the capacity to differentiate to neurons, astrocytes, and also oligodendrocytes^39^. Late descendants of RG persist in the subventricular zone (SVZ) of the lateral ventricle and the subgranular zone (SGZ) of the DG of the hippocampus, giving rise to adult neurogenesis and gliogenesis^38^. Direct evidence of hippocampal neurogenesis in adult humans remains difficult to capture and has even been questioned^40^. However, a recent study identified thousands of immature neurons in the DG of healthy individuals up to the ninth decade of life, providing direct evidence of neurogenesis in adults^41^.

The aforementioned cellular findings likely support the evidence of subfield-specific effects of exercise. In first place, physical activity induces a volume increase in the left CA subregions and shows a trend for inducing a volume increase in the left CA4/DG^42^. In another study, molecular, functional, and structural evidence from animal models indicated that exercise induces neuroplastic processes in the brain, particularly in the hippocampus^43–51^. Finally, in a recent MRI/histological study in mice, physical exercise led to an increase in gray matter volume in the hippocampal DG and CA1–3 subfields, along with an increase in neurogenesis in the DG^52^. Taken together, these results provide mechanistic insight into the neuroplastic processes in specific areas of the hippocampus that may link physical exercise to clinical improvement in SCZ.

Recent genome-wide association studies (GWASs) indicate a large genetic overlap between SCZ risk and hippocampal volume^53^. These results converge with previous evidence that a higher polygenic SCZ risk burden is associated with reduced hippocampal volumes in at-risk individuals and first-episode and chronic SCZ patients^4,54^. Moreover, previous work from our group showed that polygenic SCZ risk modulates the effect of aerobic exercise in the CA4/DG region^55^. However, little is known about the biological processes, cellular pathways, or cell types underlying the corresponding polygenic risk.

In a recent single-cell RNAseq study, Skene et al. were able to map genomic SCZ risk loci onto expression profiles of specific brain cell types; this mapping indicated that neuronal and OPC-enriched transcripts are associated with risk loci^56^. Here, we present a secondary analysis that leverages the cell type-specific expression profiles derived from the study by Skene et al. to generate cell type-specific polygenic risk score (PRS) in our sample. Given the previous postmortem evidence of decreased oligodendrocyte or OPC numbers in hippocampal subfields in SCZ^33,34^, our analysis focused on the polygenic burden associated with different stages in the development of oligodendrocytes^57,58^, i.e., RG (PRS^Rad^), OPCs (PRS^OPC^), and mature oligodendrocytes (PRS^Oli^). Our aim was to investigate whether cell type-specific SCZ PRS related to RG, OPCs, or mature oligodendrocytes are associated with volume changes in CA1, CA2/3, and CA4/DG subfields in multi-episode SCZ patients and healthy controls after 3 months of aerobic exercise.

## Patients and methods

### Participants

The sample analyzed in this study has been described in detail elsewhere^17,18^ and is the same as was previously used to determine the influence of SCZ PRS on brain structure^55^. Briefly, the original study recruited 20 multi-episode SCZ patients and 23 healthy controls in an aerobic exercise intervention group and 21 multi-episode SCZ patients in a table soccer (control intervention) group. It also included a cognitive remediation intervention for all participants. SCZ patients were recruited in the Department of Psychiatry and Psychotherapy of the University Medical Center Goettingen. Healthy controls, who had no past or current illness, were matched for age, sex, and handedness. The study protocol was approved by the ethics committee of the University Medical Center Goettingen. All participants provided written informed consent prior to inclusion in the study, and the study was conducted according to the Declaration of Helsinki. The trial is registered at www.clinicaltrials.gov (NCT01776112).

### Endurance training, table soccer

In each group, the intervention consisted of three 30-min sessions per week and lasted 3 months. Endurance training was conducted on bicycle ergometers at an individually defined intensity that was gradually increased until blood lactate concentrations of 2 mmol/l were reached, in accordance with the continuous training method (e.g., ref. ^59^). The training parameters blood lactate concentration, heart rate, and exhaustion according to the Borg scale were monitored^60^. The SCZ patients allocated to the non-endurance intervention had table soccer for the same amount of time. More details on the intervention protocols can be found elsewhere^17,18^.

### Magnetic resonance imaging acquisition

MRI data were acquired at baseline (V1) and after 3 months (V3) in a whole-body 3.0 Tesla MRI Scanner (Magnetom TIM Trio, Siemens Healthcare, Erlangen, Germany) with an 8-channel head coil. Small cushions were used between the head coil and the individuals’ heads to minimize head movements. The 3D anatomical images were acquired with a T1-weighted magnetization-prepared rapid gradient echo (MP-RAGE) sequence with a field-of-view of 256 mm and an isotropic spatial resolution of 1.0 × 1.0 × 1.0 mm³ (TR = 2250 ms, echo time = 3.26 ms, inversion time = 900 ms, flip angle 9°, number of slices = 176). All images were quality controlled by a board-certified radiologist and subsequently anonymized to blind the participants’ identities.

### Image processing

Automated hippocampal segmentation was performed with the FreeSurfer version 5.3.0 software package (http://surfer.nmr.mgh.harvard.edu). The longitudinal processing stream was used for automatic subcortical segmentation, and hippocampal subfield volumes were computed from T1-weighted images^61^. An unbiased within-subject template space and image^62^ was created by robust, inverse consistent registration^63^. Processing steps involved skull stripping, Talairach transforms, atlas registration, and spherical surface maps. Parcellations were initialized with common information from the within-subject template, which significantly increased reliability and statistical power^61^. The longitudinal processed images were used to calculate the CA1, CA2/3, and CA4/DG hippocampal subfield volumes for each participant^64^. We performed a correction for the individual intracranial volume (ICV) with the proportions method, in which each T1 volume is divided by the participant’s ICV and multiplied by the average ICV of all participants^65^.

### Genotyping and quality control

DNA from all participants was genotyped with the Infinium PsychArray (Illumina, San Diego, USA). Quality control steps (inclusion thresholds: SNP call rate >98%, subject call rate >98%, Hardy-Weinberg equilibrium >0.001, heterozygosity rate within three standard deviations) were performed with PLINK 1.9 (www.cog-genomics.org/plink/1.9/)^66^. An identity-by-state (IBS) matrix was calculated to estimate the relationship between the samples and showed that the study samples were not related.

Ancestry differences between the study participants were modeled with the EIGENSOFT package (SmartPCA) by using a principal component analysis based on a pruned subset of ~50,000 autosomal SNPs, after excluding regions with a high linkage disequilibrium^67^. All participants clustered to HapMap3 Caucasian reference populations, so none of them was excluded. We extracted the first two ancestry principal components to correct for the potential effects of population substructure in all downstream analyses.

### Imputation

Genotype imputation was performed with IMPUTE2/SHAPEIT by using its pre-phasing and imputation pipeline^68,69^. The 1000 Genomes Project dataset (Phase 3 integrated variant set) was used as the reference panel. Genetic variants with a poor imputation quality (INFO < 0.7) were removed. After all quality control steps, 20 multi-episode SCZ patients and 20 healthy controls from the aerobic exercise intervention groups and 16 multi-episode SCZ patients from the table soccer group were included in the genetic study.

### Calculation of cell type-specific PRS

Discovery sample: Summary statistics from the most recent SCZ GWAS, which was performed in a sample of 40,675 cases and 64,643 healthy controls, were used to ascertain risk variants/alleles, their p values, and associated odds ratios^70^.

Definition of oligodendrocyte lineage gene sets: The top 5% specifically expressed genes in mouse radial glia-like cells (Rad), OPCs, and mature oligodendrocytes (Oli), as published in the recent single-cell RNAseq study mentioned above^56^, constituted the gene sets used to calculate PRS specific for these cell types (Supplementary Table 1). These gene sets had a certain degree of overlap: Rad-OPC, 17.2%; OPC-Oli, 19.9%; and Rad-Oli, 3.2%.

Target sample: Three different PRS were generated exclusively on the basis of the genetic variants in the genes (±10 kb) that constitute the radial glia (PRS^Rad^), OPCs (PRS^OPC^), and mature oligodendrocyte (PRS^Oli^) gene sets. A clumping procedure was carried out (--clump-kb 500, --clump-r2 0.1) on the basis of the variants of each gene set. PRS were calculated by multiplying the imputation dosage for each risk allele by the log(Odds Ratio) for each genetic variant. The resulting values were summed to obtain an individual estimate of the cell type-specific SCZ genetic burden in each individual across ten p-value thresholds (5 × 10^−8^, 1 × 10^−6^, 1 × 10^−4^, 1 × 10^−3^, 0.01, 0.05, 0.1, 0.2, 0.5, 1).

### Statistical analyses of PRS effects on hippocampal volume changes

For each of the imaging variables under study, the baseline values (V1) were subtracted from the values at 3 months (V3), and the resulting differences were standardized. The Kolmogorov-Smirnov test found no significant deviations from a normal distribution for the hippocampal volume changes in CA1, CA2/3, and CA4/DG. The effect of PRS on volume changes in CA1, CA2/3, and CA4/DG was ascertained by univariate linear regression in R 3.5.0^71^. Age, sex, height, handedness, and the first two ancestry principal components were used as covariates in all genetic association analyses. We performed all analyses separately for SCZ patients performing aerobic exercise, SCZ patients playing table soccer, and healthy controls (who also performed aerobic exercise) to identify differences between the three groups. To address potential type I errors, we determined statistical significance after a permutation-based resampling procedure. Briefly, empirical adjusted *p* values (*P*_adj_) were determined through permutation testing of 10,000 simulations with lmPerm package^72^. These *P*_adj_ were obtained by permuting the values of the dependent variables in each of the tested models. Plots were generated with the ggplot2 package^73^.

## Results

### SCZ endurance training group

**Fig. 1:**
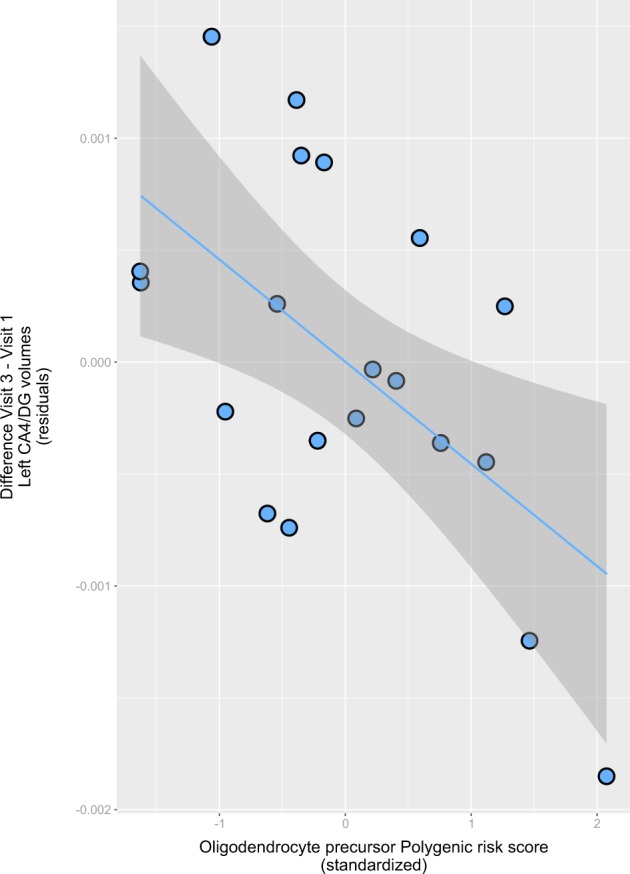
Scatterplot showing the relationship between the optimal (*p*-value threshold = 0.01) oligodendrocyte precursor polygenic score (PRS^OPC^; *x*-axis, standardized) and the change from baseline (V1) in the volume of the left hippocampal subfields CA4/dentate gyrus after 3 months of aerobic exercise (V3) (*y*-axis, corrected residuals). Positive values in the *y*-axis indicate a gain in volume after 3 months; and positive values in the *x*-axis, a higher genetic risk burden. Also shown are regression line and 95% confidence intervals based on the predicted means from the regression line

In the SCZ patients performing aerobic exercise, after correction (*P*_adj_ < 0.05) the PRS^OPC^ was significantly associated with volume changes in the left CA4/DG subfield, with an optimal threshold identified at *p* = 0.01 (Supplementary Fig. 1 and Supplementary Table 2). At all significant thresholds, high PRS^OPC^ genetic risk burden was associated with less pronounced volume increase or even a decrease over time in the left CA4/DG (Fig. 1).

**Fig. 2:**
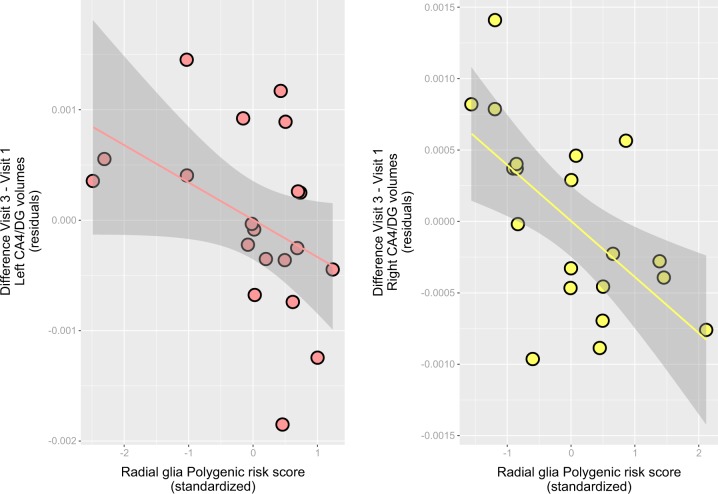
Scatterplot showing the relationship between the optimal (p-value threshold = 5 × 10^-8^ [left] and 0.05 [right]) radial glia cells-like polygenic score (PRS^Rad^; *x*-axis, standardized) and the change from baseline (V1) in the volume of the left and right hippocampal subfields CA4/dentate gyrus after 3 months of aerobic exercise (V3) (*y*-axis, corrected residuals). Positive values in the *y*-axis indicate a gain in volume after 3 months; and positive values in the *x*-axis, a higher genetic risk burden. Also shown are regression line and 95% confidence intervals based on the predicted means from the regression line

PRS^Rad^ analysis in this group showed a similar direction of the genetic effects, but in this case changes associated with genetic load were observed in both the left and right CA4/DG subfields (Supplementary Fig. 1 and Supplementary Table 2). The optimal thresholds were 5 × 10^−8^ (left) and 0.05 (right), and a high PRS^Rad^ genetic load was associated with a less pronounced volume increase or a decrease after exercise in left and right CA4/DG subfields (Fig. 2).

The study of the effects of PRS^Oli^ did not show any consistent effects in any of the subfields analyzed (Supplementary Figs. 1–3). Likewise, PRS^OPC^ and PRS^Rad^ did not influence volumes changes in the CA1 or CA2/3 subfields (Supplementary Figs. 2,3).

### Healthy controls endurance training group

In the group of healthy controls performing the exercise intervention, the only polygenic effect observed was an influence of PRS^Rad^ on volume changes after exercise in the left CA1 and left CA2/3 regions at several thresholds (*P*_adj_ < 0.05) (Supplementary Figs. 2, 3). In both left subfields, a high PRS^Rad^ genetic load was associated with a less pronounced volume increase or a decrease after exercise (data not shown).

### SCZ table soccer group

In the group of SCZ patients performing the control intervention (table soccer), we found no consistent polygenic effects for any of the PRS in any of the hippocampal subfields (Supplementary Figs. 1–3). The only effects observed were single-threshold associations of PRS^OPC^ with left and right CA4/DG volume changes, with opposite directions in the left and right hemispheres.

## Discussion

Our results suggest that the beneficial effects of exercise in SCZ patients might be modulated by cell type-specific differential polygenic risk. Our approach builds upon previous studies by our group: in brains from SCZ patients, we observed a reduced number of oligodendrocytes in the left CA4 region in stereological postmortem studies^33,34^ and, in the same samples, showed a reduction (*p* < 0.10) in the density of oligodendrocyte transcription factor (OLIG)1-positive cells by immunohistochemistry^35^. OLIG1-specific antibodies are known to stain precursor forms and mature oligodendrocyte populations, and OLIG1 is needed for progenitor development and repair of myelin^74^. The above findings led us to hypothesize that the decreased number of oligodendrocytes in the left CA4 region indicates a disturbed regenerative process^75^.

Our group was the first to establish the role of SCZ PRS in changes in left hippocampal subfields after sustained aerobic exercise^55^. Here, we extend these results and show that SCZ polygenic risk for certain cell types of the glial/oligodendrocyte lineage exerts a modulatory effect on the CA4/DG volume changes promoted by exercise. In our study, polygenic risk associated to mature oligodendrocytes does not have any influence on these effects. This suggests that the mechanism of action of these genetic modulatory effects likely involves neuroplastic processes involving gliogenesis (and potentially neurogenesis), or that at least these effects are more important that the ones related to mature oligodendrocytes. Of note, a recent study in an animal model of toxin-induced demyelination showed that exercise enhances oligodendrogenesis and remyelination and increases the proportion of remyelinated axons^76^.

OPCs derived from RG cells display a widespread distribution in mammalian brains and serve as a source of myelinating oligodendrocytes. Recent studies have provided compelling evidence that immature OPCs can differentiate to myelinating OPCs^77,78^. They likely represent the cellular substrate underlying different forms of adult plasticity and form a homeostatic network capable of reacting to many types of injury^79^. Moreover, it is known that OPCs and oligodendrocytes not only generate myelin but also provide trophic support to axons of principal neurons^80,81^. Also, parvalbuminergic interneurons are myelinated in the cortex and hippocampus of mice and humans, and a dysfunctional cross-talk between these cells and oligodendrocytes has been suggested to contribute to the cellular pathology of SCZ^82^. It may thus be possible that disturbed oligodendrocyte development leads to dysfunctions in interneurons, which is a highly replicated finding in SCZ^83^.

On the basis of our findings, we hypothesize that high PRS^OPC^ and PRS^Rad^ interfere with neuroplastic changes triggered by aerobic exercise in SCZ patients and that these cell-specific PRS may lead to failed regeneration mechanisms in the hippocampus. An important proportion of the variation on the hippocampus volume change triggered by exercise is explained by these polygenic estimates (optimal *R*² ~ 0.40 for PRS^OPC^ and PRS^Rad^). However not all variation is explained by them and further studies are warranted to characterize the contribution of polygenic risk associated to neurons, astrocytes or microglia to such volumetric changes in these patients.

We could show that these effects are dependent on the disease status and the type of intervention. Our data indicate that in healthy controls PRS influence the effects of exercise in CA1 and CA2/3 subfields, but not in CA4/DG. Moreover, we did not detect an effect in SCZ patients playing table soccer (control intervention), supporting the notion that the corresponding PRS are relevant for the effects of aerobic exercise on brain structure.

Our results suggest that genetics may shed some light on the conflicting evidence of the effects of aerobic exercise on hippocampal volume and cognitive function in SCZ^16–18,24,26,27,84^. Here, the individual load of SCZ genetic risk seems to modulate the effects of aerobic exercise. This genetic risk-driven modulation fits with the evidence of high heritability for the total hippocampus and its subfields^85–87^ and with a recent study showing a clear overlap between genetic factors related to SCZ risk and hippocampal volume^53^. Moreover, a recent study of brain imaging phenotypes that used the UK Biobank cohort found that genes associated with brain development and plasticity tend to be associated with mental disorders, including SCZ and severe depression, while genes coding for iron-related proteins tend to be associated with neurodegenerative diseases, such as Alzheimer's disease^88^.

Our study also has some limitations. First, the modest sample size of the original study warrants replication of our findings in independent samples of SCZ patients who perform the same aerobic exercise intervention protocols and are assessed with the same instruments. Second, a randomization procedure was not used to allocate the SCZ patients to the endurance training augmented with cognitive remediation or table soccer augmented with cognitive remediation group, which may have led to potential selection bias and baseline differences in psychopathology and dose of antipsychotic medication^17,18^. Third, our exploratory analyses could not detect any effect of PRS on psychopathology, cognition or functioning in our samples (data not shown), probably due to a limited sample size with low power to detect these genetic influences on behavioral outcomes. Finally, in order to confirm the relatively high *R*^2^ estimates in the present study, replication studies are warranted in larger and independent samples.

We conclude that a high polygenic burden may influence neuroplastic processes in the hippocampus during aerobic exercise in SCZ. We propose a gene × environment interaction in which the genetic load influences the effects of the intervention on neuroplastic processes via dysfunctions in RG and OPCs. Identifying the cell types that drive clinical improvement during aerobic exercise will provide mechanistic insight into the underlying biological processes that direct hippocampal plasticity.

## Supplementary information

Supplementary
