# Supplementary material for: Polygenic burden associated to oligodendrocyte precursor cells and radial glia influences the hippocampal volume changes induced by aerobic exercise in schizophrenia patients
Source: Transl Psychiatry. 2019 Nov 11;9:284. doi: 10.1038/s41398-019-0618-z (PMC6848123; doi:10.1038/s41398-019-0618-z)
Supplement: Supplementary file 1 — Supplementary [file 41398_2019_618_MOESM1_ESM.docx]

**Supplementary Information:**

**Polygenic burden associated to oligodendrocyte precursor cells and radial glia influences the hippocampal volume changes induced by aerobic exercise in schizophrenia patients**

Sergi Papiol, PhD^1,2#^, Daniel Keeser, PhD^1,3^, Alkomiet Hasan, MD^1^, Thomas Schneider-Axmann, MSc^1^, Florian Raabe, MD^1,4^, Moritz J. Rossner, PhD^1^, Heike Bickeböller, PhD^5^, Ludovico Cantuti-Castelvetri^6^, Mikael Simons^6,7,8^, Thomas Wobrock, MD^9^, Andrea Schmitt, MD^1,10^, Berend Malchow, MD^1^*, Peter Falkai, MD^1^*

^1^Department of Psychiatry, University Hospital, Nussbaumstrasse 7, 80336 Munich, Germany

^2^Institute of Psychiatric Phenomics and Genomics (IPPG), University Hospital, Ludwig Maximilian University, Nussbaumstrasse 7, 80336 Munich, Germany

^3^Institute of Clinical Radiology, Ludwig Maximilian University Munich, Marchioninistrasse 15, 81377 Munich, Germany.

^4^International Max Planck Research School for Translational Psychiatry, Kraepelinstr. 2-10, 80804 Munich, Germany

^5^Department of Genetic Epidemiology, University Medical Center, Georg-August-Universität Göttingen, Humboldtallee 32, 37073 Göttingen, Germany

^6^German Center for Neurodegenerative Diseases (DZNE), Feodor-Lynen Str. 17, 81377 Munich

^7^Munich Cluster for Systems Neurology (SyNergy), 81377 Munich, Germany

^8^Institute of Neuronal Cell Biology, Technical University Munich, 80805 Munich, Germany

^9^Department of Psychiatry and Psychotherapy, County Hospitals Darmstadt-Dieburg, Krankenhausstraße 7, 64823 Groß-Umstadt, Germany

^10^Laboratory of Neuroscience (LIM27), Institute of Psychiatry, University of Sao Paulo, Rua Dr. Ovídio Pires de Campos 785, Sao Paulo-SP 05403-903, Brazil

*Authors contributed equally


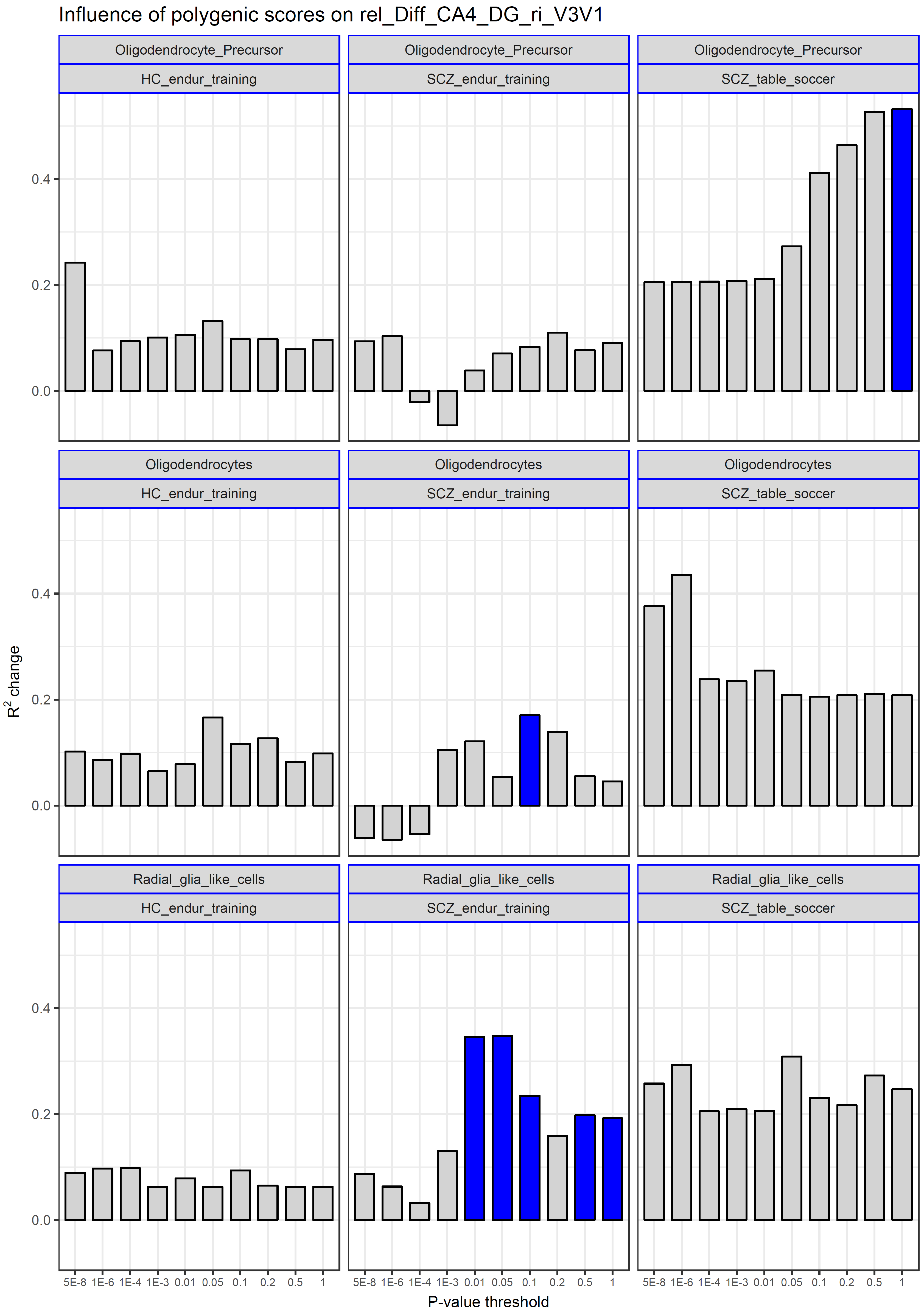
**Supplementary Figure 1.** Analysis of the association between cell type-specific polygenic risk scores (radial glia, PRS^Rad^; oligodendrocyte precursor cells, PRS^OPC^; and mature oligodendrocytes, PRS^Oli^) and volumetric changes between baseline (V1) and 3 months (V3) in the left CA4/dentate gyrus (DG; left figure) and right CA4/DG (right figure) in the 3 subgroups included in this study. All results are corrected for sex, age, height, handedness, and 2 ancestry principal components. The x-axis shows the ten p-value thresholds (5E-8 through 1) analyzed, and the y-axis indicates the gain in the amount of variance explained by PRS, measured with R^2^. Blue bars indicate thresholds of PRS, with P_adj_<0.05.


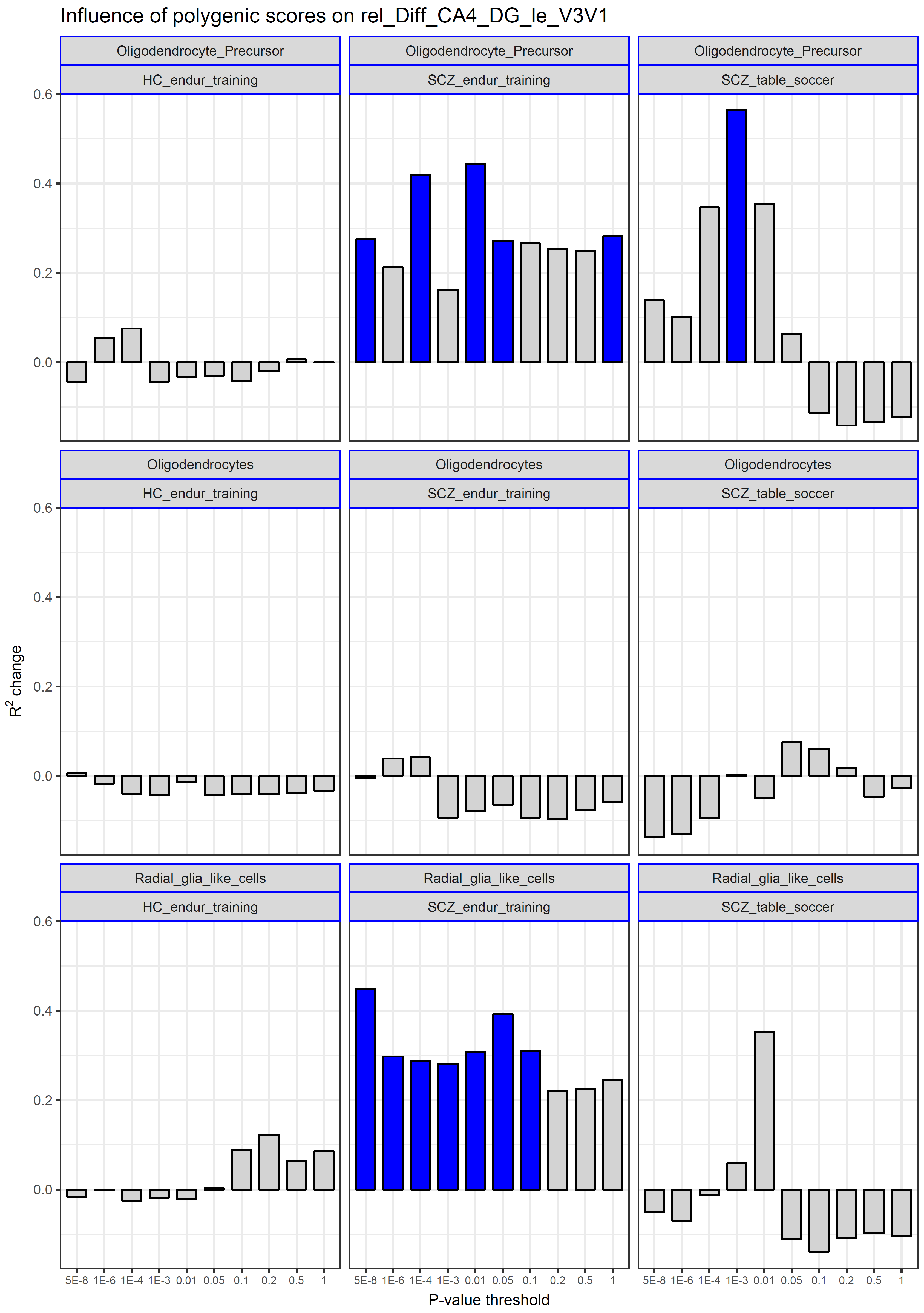


**Supplementary Figure 2.** Analysis of the association of cell type-specific polygenic risk scores (radial glia, PRS^Rad^; oligodendrocyte precursor cells, PRS^OPC^; and mature oligodendrocytes, PRS^Oli^) with volumetric changes between baseline (V1) and 3 months (V3) in the left CA1 (left figure) and right CA1 (right figure) in the 3 subgroups included in this study. All results are corrected for sex, age, height, handedness, and 2 ancestry principal components. The x-axis shows the ten p-value thresholds (5E-8 through 1) analyzed, and the y-axis indicates the gain in the amount of variance explained by PRS, measured with R^2^. Blue bars indicate PRS thresholds with P_adj_<0.05.


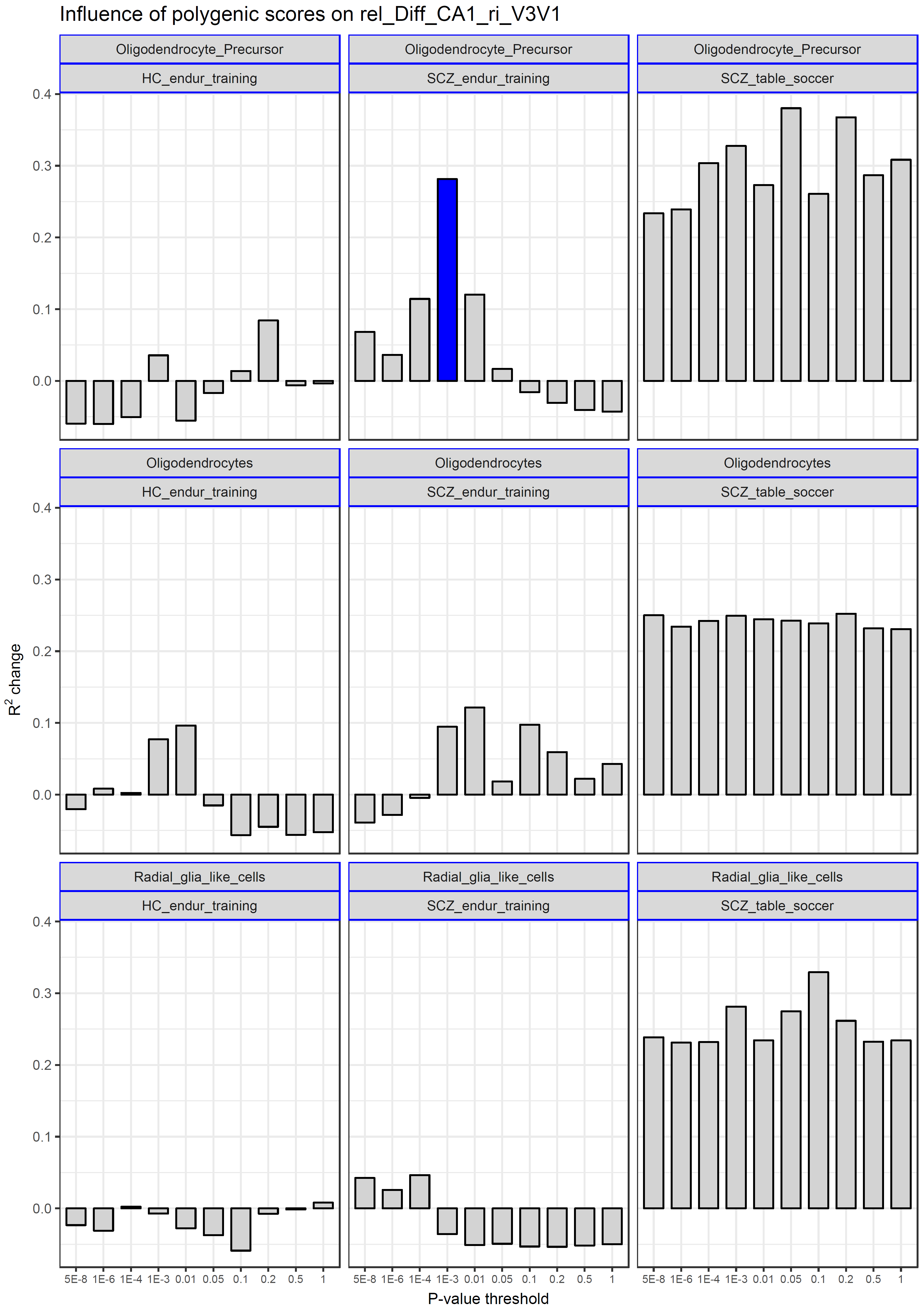

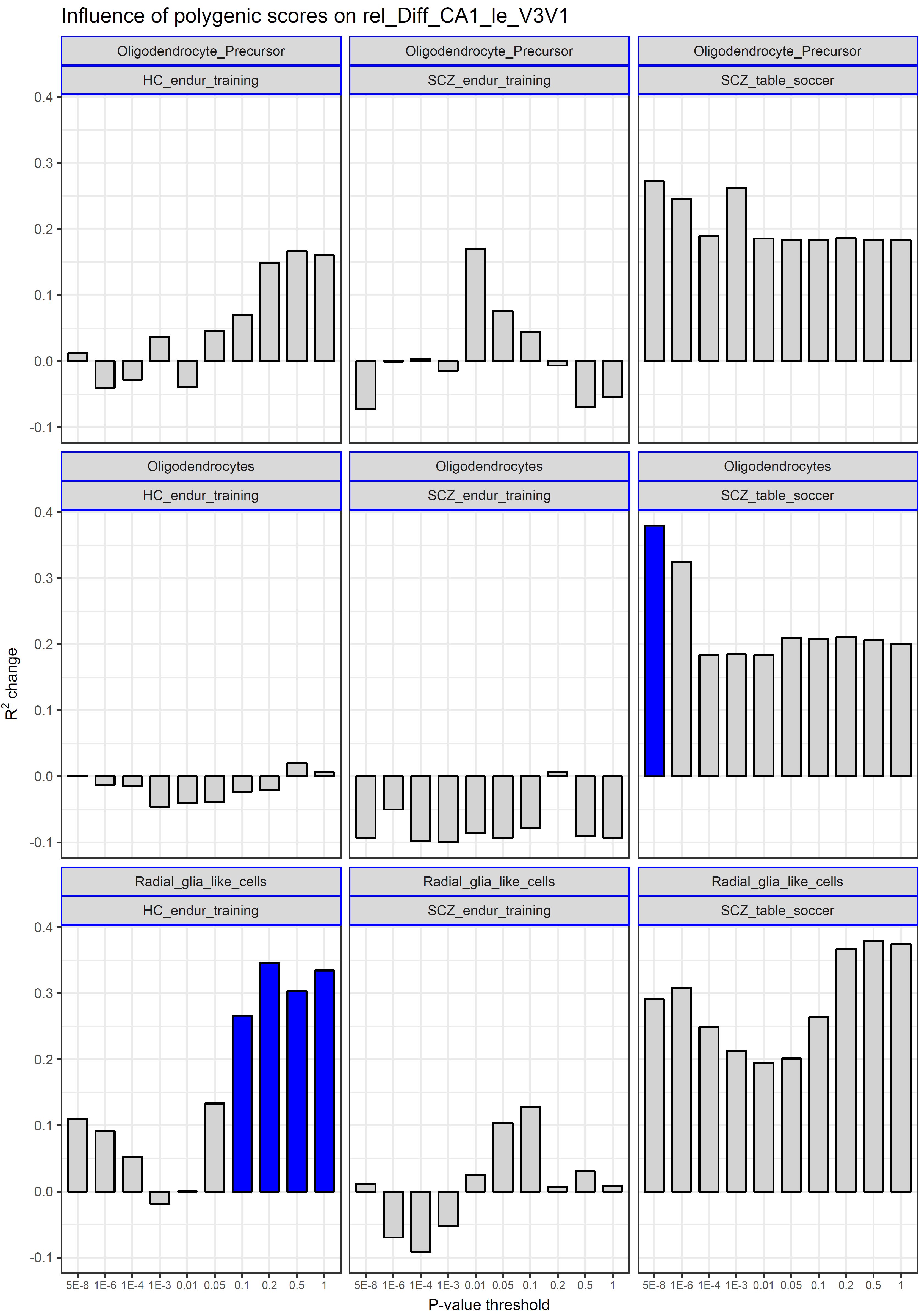


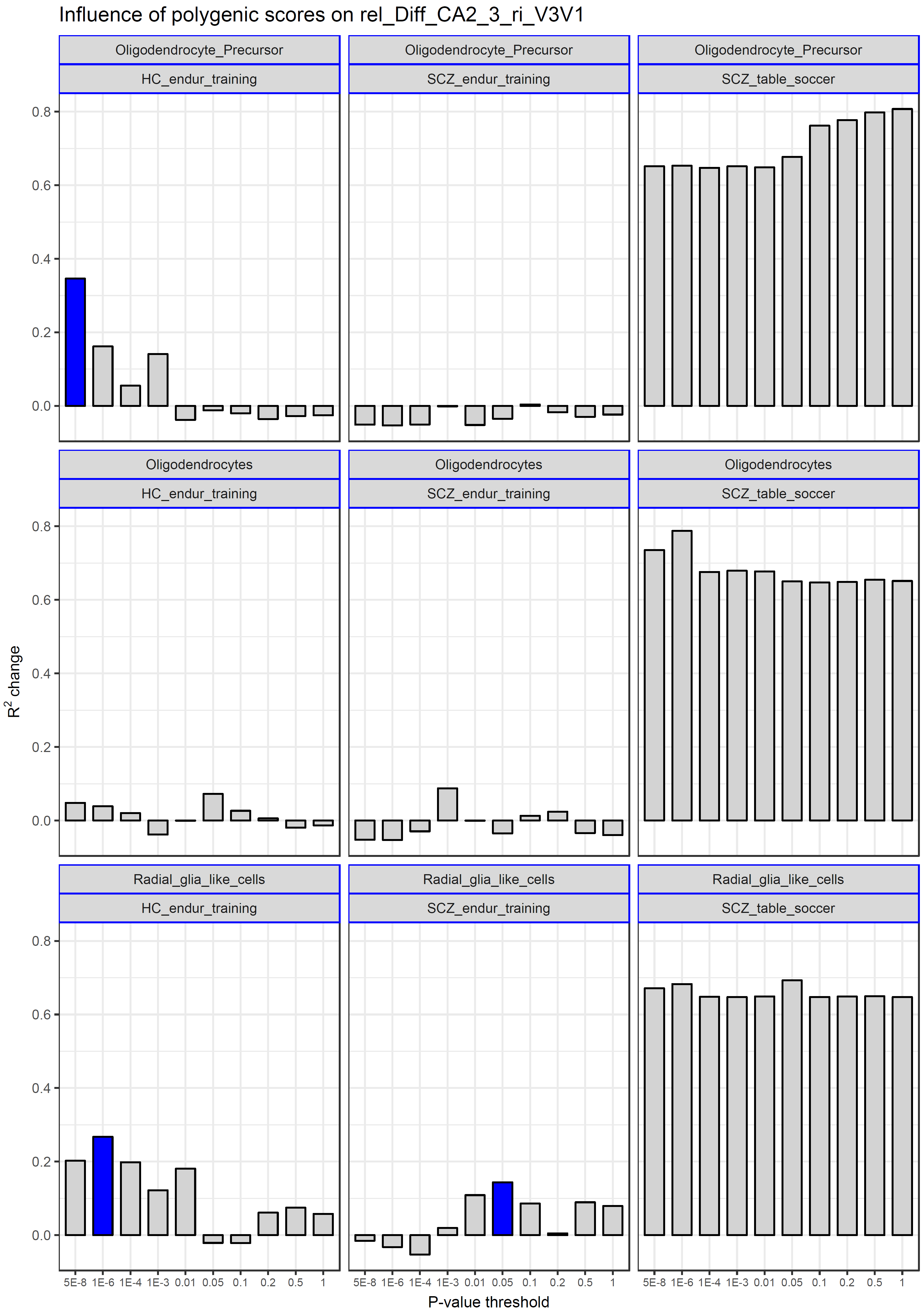
**Supplementary Figure 3.** Analysis of the association of cell type-specific polygenic risk scores (radial glia, PRS^Rad^; oligodendrocyte precursor cells, PRS^OPC^; and mature oligodendrocytes, PRS^Oli^) with volumetric changes between baseline (V1) and 3 months (V3) in the left CA2/3 (left figure) and right CA2/3 (right figure) in the 3 subgroups included in this study. All results are corrected for sex, age, height, handedness, and 2 ancestry principal components. The x-axis shows the ten p-value thresholds (5E-8 through 1) analyzed, and the y-axis indicates the gain in the amount of variance explained by PRS, measured with R^2^. Blue bars indicate PRS thresholds with P_adj_<0.05.


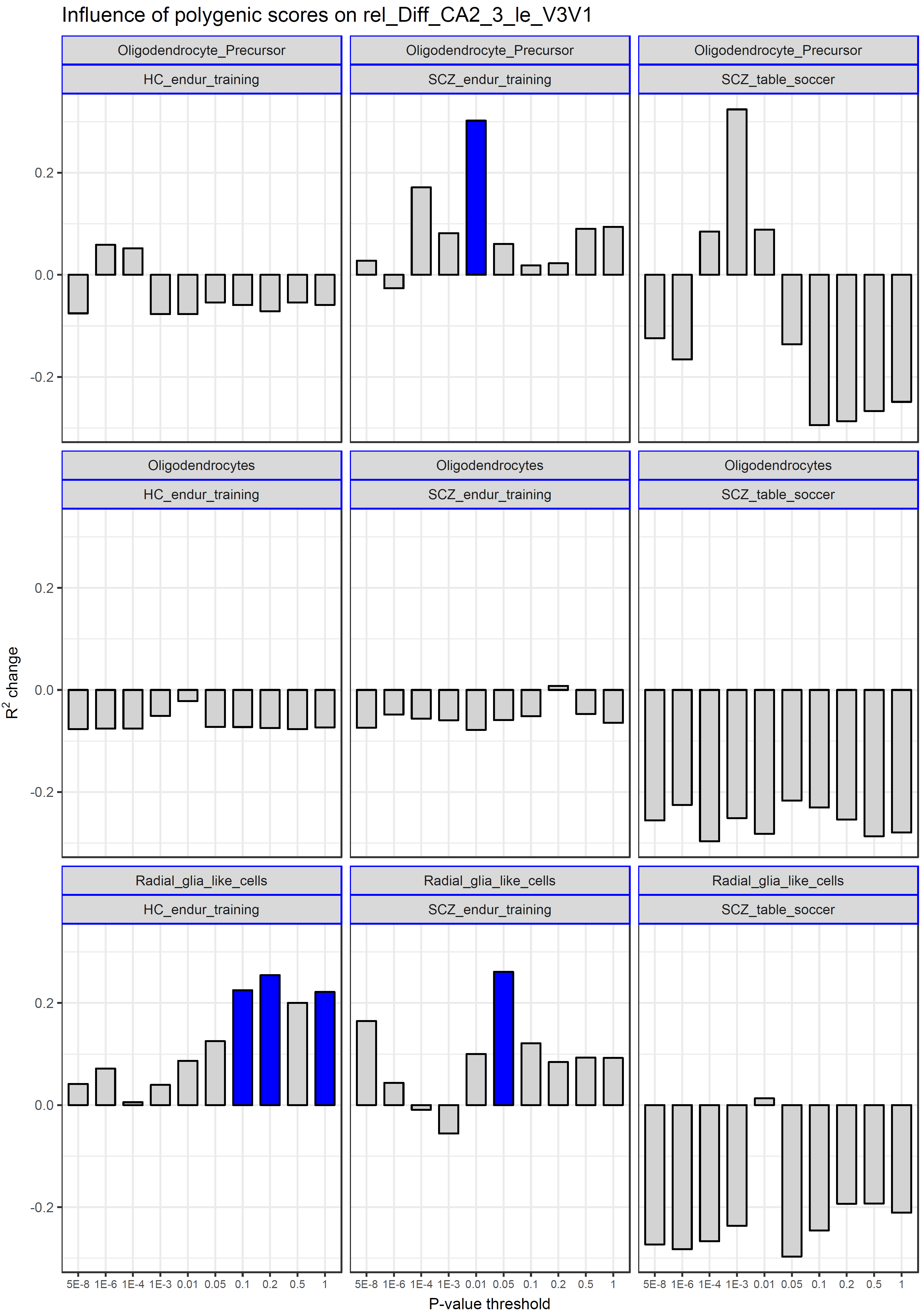


**Supplementary Table 1.** Table summarizing the top 5% specifically expressed genes in mouse oligodendrocyte precursor cells, mature oligodendrocytes, and radial glia-like cells, as published in a recent single-cell RNAseq study (Skene et al., 2018). Genetic variants on the human homologous of these genes constitute the basis for the calculation of schizophrenia polygenic risk scores for radial glia (PRS^Rad^), oligodendrocyte precursor cells (PRS^OPC^), and mature oligodendrocytes (PRS^Oli^).

| **Cell type** | **5% most specific transcripts** |
| --- | --- |
| Oligodendrocyte precursor cell | Knl1, Gsx1, Tmem255b, Matn4, Fam89a, Neu4, Cspg4, Carmil3, Pdgfra, Lad1, Pmel, Shc4, Gpr17, Cacng4, Selenoh, Rlbp1, 0610040J01Rik, Snx22, Olfr222, Emid1, Bex3, Grk2, Scel, Twnk, Grk3, 5730559C18Rik, Angptl1, Miga1, Olig2, Rbpjl, Chst5, Sox10, Sapcd2, Rnf43, Susd5, Zfp488, Vcan, Kmt5c, Mia2, Spout1, Bsx, Afap1l2, S100a3, Adam12, Tmem100, Kmt5b, Caskin2, Trdn, Selenow, Calcrl, Garem2, Lhfpl3, Olig1, Sox8, C1ql1, Sh3bp4, Spry4, Sema3d, Ints6l, Mmp15, G0s2, Pcdh15, Meox1, Afdn, Galnt3, H1foo, Selenoi, Hikeshi, Garem1, Etv4, Prune1, Ndufaf8, Xylt1, Tnr, Mylk3, Bricd5, Ubash3a, Tepsin, Selenof, Zcchc24, Naxd, Ly6g6d, Ptprz1, Ascl1, Arhgap31, Ccnd1, Dct, Atp6v1g3, E2f8, Selenot, Bmp4, Fbxo43, Gpnmb, S100a4, Gsap, Grid2ip, Rgcc, Miga2, Matn1, Gucy2f, Carmil1, Snx9, Sox3, S100a16, Selenon, Mov10l1, Pllp, S100a1, Adamtsl3, Slc36a3, Bcan, Prkg2, Bcas1, Prrx1, Itpr2, Kcnip3, Sox6, Tmem200b, Scrg1, Chst3, Chst7, Nkx2-2, Aspscr1, Kcnj16, Dmc1, Cav1, Mmp2, Eomes, Spry1, Col16a1, B3gnt7, Krt1, Tcf19, Pstpip2, Selenok, Nmrk2, Tnfrsf11b, Dusp9, Prr11, Frmd7, Cyp2j6, Abca6, Ppox, Gas1, Efs, Kank1, Amz1, Abhd2, Gm2a, Pxdc1, Mrpl58, Pcdhgc3, Lima1, Ppfibp1, Mif4gd, Loxl3, Ung, Dmrta1, Itgav, Ccdc114, Ednrb, Wipf1, Chaf1b, E2f7, Ostf1, Prkcq, Mtss1l, Nfatc1, Creb5, Ruvbl2, Mapk7, Tnfrsf13c, Selenom, Selenoo, Rhbdl2, 4930427A07Rik, Uhrf1, Cnga2, Orai1, Asl, Galnt10, Cd9, Traf4, Mfsd2a, Lims2, Epn2, Col11a2, Fignl1, S1pr2, Lmcd1, Dmrt3, Rapgef3, Nr2e1, Brip1, Cyp3a13, Hoxb8, S100a13, Abtb2, Lpcat2, Adra2b, Selenos, Cspg5, Car8, Kif7, Henmt1, Eya1, Tgfa, Agbl2, Fzd9, Ltbp1, Sox21, Plpp4, Hapln3, Fam114a1, Gpt2, Cdk2, Map3k1, Chadl, Timp4, Slc6a19, Fam221b, Zc3h3, Myb, Hoxa7, Nrm, Megf11, Gpc5, Slc13a5, P2rx7, Ptk7, Limd1, Itga9, Arhgef19, Mcm9, Pcdhb10, Chst11, Notch1, Gsg2, Ampd3, Cdh26, Clic3, Tril, Sulf2, Rep15, Sapcd1, Dll1, Ccdc70, Svil, Slc43a1, Gpr37l1, Cabp4, Dpyd, Stom, Teddm1b, Fgfrl1, Ticam1, E2f2, Smoc1, Kif14, Cr2, Ugdh, Chpt1, Pglyrp1, Fgf5, Gjc3, Maz, Abcb4, Rsg1, Fam69c, Fut10, Arsb, Il17d, Myt1, Fam19a4, Tlcd1, Flt3l, Ogg1, Grb7, Sycn, Txndc12, Spin4, Tap1, Hey2, Fbxo7, Col5a3, Iqgap3, Arhgap22, Usp24, Gpr171, Ncapg2, Pcdhga11, Neil3, Evc, Erbb3, Dock1, Serinc5, Col9a3, Dppa4, Ffar2, Sowahc, Rnf225, Elfn2, Mcm5, Slc12a4, Mkrn2os, Socs1, Polg, Slc25a15, Hip1, Tlr5, Sema5b, Apobec3, Tns3, Duox1, Serpine2, Nlrp6, Atp13a5, Sall3, Mcm2, Adora2b, Lrp2bp, Gimap1, Tmem144, Fzd8, Uts2r, Zfp36l2, Prdm5, Myod1, Gm7173, Mfsd13a, Acp7, Sstr1, Arap1, Sh3d19, Nemp2, Plxnb3, Pla2r1, Grtp1, Brca1, Glis3, Adamts6, Igfbp3, Pcdhgb7, Ada, Rab20, Ccl22, Dnah11, Slc9b1, Dscc1, Snx33, Dnase1l2, Rnf180, Ncmap, Cdkn2c, Bambi, Gal3st1, Rasa3, Slc24a1, Itpkb, Pcdhga8, Rasl12, Adamts1, Card11, Ush2a, Lrp4, Cenpi, Lhfpl2, Nectin4, Pcdhga1, Cdc6, Metrn, Fanca, Rftn2, Pkd2l1, Serpind1, Ctse, 2610020H08Rik, Antxr1, Epha2, Prickle3, 4921536K21Rik, Ankmy1, Tnfrsf1a, Ccdc134, Zeb1, Slc22a13, Scfd2, Dll3, Phlda1, Atp8b1, B3gat2, Mms22l, Pak4, Cyb5r2, Nckap5, Dbx2, Ska3, Bdkrb1, Itgb7, Mcidas, Pold1, Dmbt1, Pde4c, Hjurp, Trpv4, Gnat2, Tfpt, Fbn2, Ccdc18, Duox2, Mcm3, 4930579G24Rik, Prex1, Mki67, Itgb8, Tor3a, Cdc42ep1, Ddx58, Mcm7, Bmp7, Cdk5rap2, Tmem176a, Mcm10, Rab31, Ptpn14, Tg, Cdo1, Bard1, Tst, Foxm1, Cdt1, Lrp1, Lnx1, Fam229a, Cdca2, Mybpc1, Lrrn4cl, Slc46a3, Tap2, Nhsl1, Lmntd1, Nlgn2, Lexm, Kif20b, Rin3, Zfat, Rhbdf1, Adam9, Socs3, Nfia, Sox5, Fadd, Msrb3, Syde1, Epas1, Fam209, Pld1, Kif13a, Pstpip1, Cmtm3, C330027C09Rik, C1qtnf5, Nsl1, Nptxr, Pole, Prkd3, Hcst, Kif19a, Asap3, Klhl5, Murc, Loxl4, Ppp1r18, Clspn, Ankle1, Il15ra, Cdc14a, Cytip, Eid3, Spry2, Acot2, Myh2, Cdca7l, Mtmr11, Zfp36l1, Plekhn1, Kif18b, Osgin2, Cenpm, Rad54b, Hepacam, Nckap5l, Raver1, Rin2, Guca1a, Diaph3, Adm, Kcnrg, Zhx2, Phldb1, Nmi, Zwilch, Esco2, Cyp4f13, 3632451O06Rik, Lpar3, Recql4, Stil, Jam2, Dock9, Plppr5, Ppp3r2, Fmnl3, Fosb, Plscr4, Plekha2, Lrrn1, Gltp, Col20a1, Cobll1, Trim25, Dscam, Mks1, 2810459M11Rik, Lrguk, Il1rap, Tnfaip6, Mtfr2, Ankrd28, Ccdc88c, Ackr3, Exoc3l, Kcnh8, Sigirr, Pole2, Sept8, Chek1, Fshb, Acap1, Fzd4, Aebp1, Plcb3, Ptgir, Npc1, Wscd1, Adamtsl5, Cav2, Nfatc2, Kif15, Amotl2, Polg2, Bcl6, Mob1a, Adamts18, Ccbe1, Spon1, Tagln2, Dtl, Hgf, Sebox, Krcc1, Prkd1, Eps8l1, Bcl6b, Col27a1, Mcm4, Mark2, 6430573F11Rik, Vgll4, Nt5e, Hmox1, Morc4, Mmp16, Tnfsf12, Ltb, Sdc3, Kif11, C1qtnf6, Slc34a3, Aspm, Ccdc78, Cacng8, Fads2, Ikzf2, H2-Ob, Gab1, Dnmbp, Inhbb, Pdpn, Tmem176b, Stard13, Mei1, Asf1b, Fam198a, Smox, Midn, Eri1, Suclg2, Ssfa2, Pde6c, Stk32a, Kdm8, Slc38a3, Cmtm4, Efna1, Sis, Sema5a, Dpf3, Incenp, Cpxm1, Rhoc, Tgfbr3l, Ring1, Mmp14, Psrc1, Dgka, Zfp169, Mcur1, Sbspon, Ltbr, Ddx11, Alpl, Kcnj10, Lrp5, Luzp2, Slc2a9, Sept10, Nacc2, Hadha, Ttf2, Qpct, Rtkn2, Tmco4, Hip1r, Lingo4, Rcc1, Sp6, Slc25a37, Lgr5, Oplah, Frmpd1, Arhgap9, Wfikkn1, Sorbs3, Qk, Espl1, 3110035E14Rik, St14, Wdr62, Man2b2, Frzb, Kif4, Pias4, Mpzl1, Syde2, Ttk, Slc26a2, Mro, Cdca7, Ehd2, Ace2, Atp6v0e, Gpd1, Sox2, Hist1h2bb, S100b, Asrgl1, Maff, Ifit2, Rarg, Ccnd3, Hspa1l, S100a6, Pcsk6, Col10a1, Fam117a, Acss1, Evpl, Thbs4, Tmc4, Rnf144a, Rbm20, Pid1, P2ry14, Troap, Chaf1a, Hnf1b, Il17rd, Ier2, Avil, Csnk2b, Bicc1, Psmb8, Fanci, Igsf21, Trib1, Ninj2, Wdr90, Ncapd2, Cryl1, Nat2, Pcolce2, Carnmt1, Ppp1r14b, Nfkbie, Prdm9, Lamc1, Kcnk10, Nfkb1, Mettl8, Amigo3, Snai2, Anpep, Ebf4, Mtmr10, Nkd1, Vegfa, Naif1, Phlpp1, Dscaml1, Timeless, Pcgf1, Mcm8, Prtg, Abhd4, Nek8, Ss18, B3glct |
| Mature oligodendrocyte | Hoxd8, Klk6, Hapln2, Hoxb8, Gjb1, Selenoi, Selenop, Mog, Selenok, Cldn14, Cldn11, Mobp, Tst, Ermn, Ugt8a, Trf, Tmem125, Aspa, Opalin, Tmem88b, Plp1, Cnp, Ppp1r14a, Tll2, Mrpl58, Garem1, Sec14l5, Nipal4, Serpinb1a, Ints6l, Adamts4, Garem2, Atp10b, Slc34a3, Ninj2, Pdlim2, Mal, Mbp, Gsn, Cyp3a13, Gjc2, Mag, Pkd2l1, Fa2h, Slc45a3, Efhd1, Rab37, Itgad, Selenot, Galnt6, Car14, Erbb3, Miga1, Il23a, Insc, Selenos, Elovl7, Cryab, Hoxd1, Gpr37, S1pr5, Sgk2, Enpp6, Bfsp2, Elovl1, Tspan2, 1700001P01Rik, Cmtm5, Pllp, Plxnb3, Kmt5c, Selenow, Selenof, Selenom, Gjc3, Cpm, Mia2, Cdh19, Ly6g6d, Sept4, Smco3, Car2, Gng8, Hikeshi, Slpi, Itgb4, Ankub1, Miga2, Plekhh1, Myrf, Evi2a, Pla2g4a, Slc44a1, Gltp, Naxd, Pla2g16, Prune1, Tmem63a, Anln, Sp7, Grb14, Opn4, Prr5l, Tmbim1, Tnfaip6, Arsg, Gamt, Pde8a, Gal3st1, Adamtsl4, Plekhg3, Ndufaf8, Selenoo, Sis, Rhog, Ttyh2, Frmd8, Cd82, Acy3, Unc5b, Slc5a11, Twnk, Hoxa7, Tspan15, Lpar1, Rab7b, Tprn, Dock10, Kcnk13, Nkx6-2, Tnni1, Cers2, Pls1, Grk2, Snx33, Arhgef10, Enpp2, Hoxa4, S100b, Myo1d, Il12rb1, Bcas1, Cyp27a1, Litaf, Gpr62, Tmem98, Csrp1, Afdn, Wfikkn2, Ly6g6f, Plekhb1, Dbndd2, Fam57a, Carns1, Bex3, Dock5, Gramd3, Gatm, Klhl4, H2-Ab1, Ndrg1, Carmil1, Padi2, Ldlrap1, 4933413G19Rik, Casr, Mcam, Arpc1b, Sox10, Tnfrsf13c, Slc12a2, Cdc42ep1, S100a16, Gp2, Tubb4a, Cnksr3, Mob3b, Wnt3, Gna12, Kif19a, Gab1, Hoxb9, Sirt2, Prkcq, Olig1, Selenoh, Kmt5b, Itgax, Npsr1, Ppfibp2, Pigz, Fgfr2, Srpk3, Srd5a1, Sema4d, Angpt4, Slain1, Josd2, Olfml1, Dapk2, Nkx2-9, Lgi3, Btbd16, Rffl, Creb5, Piga, Card19, Esrp1, Sh3tc2, Rnf13, Npb, Tfeb, Prrg1, Atp8b1, Adssl1, Cacna2d4, Stxbp3, Spout1, Rtkn, Serinc5, Emilin2, Zfp488, Qdpr, Trim59, Sypl, Map6d1, Phldb1, Hcn2, Phlda3, Rtkn2, Kcnj10, Slc4a2, Col9a3, Cpox, Abhd4, Il1rap, Clic4, Cdc42ep2, Tmem123, Arhgef19, Otud7b, Desi1, Ccdc13, Zdhhc9, Tmeff2, Jam3, Nfasc, Slc48a1, Ddr1, Mboat1, Olig2, Tmprss5, Alox5, Kcnk5, Tjap1, Micall1, Map7, Qk, Kndc1, Ppp1r16b, Tesk2, Plin3, Tmcc3, Nod1, Eml1, D16Ertd472e, Gng12, Pstpip2, Carmil3, Tepsin, Slc6a9, Lims2, Hhip, Dusp15, Tmem141, Sspo, Bace2, Adi1, Rhpn2, Ado, Rab44, Secisbp2l, Gng11, D7Ertd443e, Wscd1, Xrcc3, Hist1h2bc, Tmem81, Plekhg1, Reep3, Arhgap23, Prr18, Rap1a, Fah, Chst3, Pacs2, Cd9, Ano4, Spsb1, St6galnac3, Dmrta1, Kank1, Col11a2, Sept8, Tmod1, Itgb7, B3gnt9, Ccp110, Fnbp1, Zbtb7b, Gss, Sytl2, Dusp10, Enpp4, Psat1, Slc25a38, Tmc6, Plekhf1, Chn2, Tmem229a, Smtnl2, Prima1, Plekha1, Fth1, Ypel2, Taldo1, Polr3e, Shisa4, Usp54, Apod, Rhou, Pacsin3, Dusp16, Ctsk, Gpr17, Dct, Cntn2, Syngr2, Aatk, Smad7, Fam83d, Serpind1, Npc1, Lap3, St18, Chdh, Rnf122, Agmo, Hs3st1, Hes3, B3galt5, Abca2, Fads6, Selenon, Pmp22, Daam2, Tgfa, Nfe2l3, Dip2a, Sox8, Hepacam, Tsc22d4, Myo6, Pcyt2, Pex5l, Efna1, Tjp2, Sema6a, Sept1, Npc2, Wipi1, Dpy19l1, Bche, Efcab14, Cela1, Erbin, Lrrn1, Mtmr10, Metrn, Hist2h3c1, Lss, Il17rb, Fgfrl1, Gpd1, Rnf130, Golga7, Hist1h4h, Aldh3b1, Inppl1, Phlpp1, Col20a1, Tns3, Ptgds, Hadh, Nkain2, Fam102a, Rnf135, Hmgcs1, Plat, Kat2b, Llgl1, Sh3gl3, Gstm7, Cd81, Sspn, Rinl, Ctnna3, Abhd17b, Tmem159, Fbxo36, Synj2, Gabrr2, Rab31, Vamp3, Prob1, Plod1, Myo1e, Shisa8, Nmral1, Bmf, Fbxo32, Matn1, Abtb2, Ldlr, Acss2, Wipf1, Cyp51, Il33, Rbpjl, Pld1, Rnf141, Pkd2l2, Entpd5, Erp27, Fhl3, Scarb2, Kcna1, Clgn, Tyro3, Slc25a45, Abhd5, Itgb8, Kif13b, Cdk18, Magt1, Clmn, Aif1l, Pcdh9, Cyp2j6, Plpp2, Fam234a, Gprc5b, Eya2, Trim13, Nipa1, Arrdc2, Parvb, Hist1h1c, Grk3, Sorbs3, Gsto2, Slc29a3, Epb41l3, Ick, Cdk19, Rassf10, Lpcat2, Smim1, Dhcr24, Pcsk6, Creb3l2, Degs1, Bpgm, Sema3d, Strn, S100a13, Crybg3, Phgdh, Kctd11, Tppp, Efnb3, Tmem189, Sc5d, March8, Nde1, Mif4gd, Agpat4, Itpr2, Ccnyl1, Pip4k2a, Hist1h2bl, Ctps, Thbs3, Barx2, Hist1h2be, Paqr8, Arap1, Trp53bp2, Pcolce2, Megf10, Scrg1, Lamp1, Rell1, Tradd, Depdc1b, Plcl1, Lad1, Tmem151a, Fez1, Snx9, Sbf1, Hacl1, Fam214a, Cyp20a1, Ell2, Nkd1, Fam53b, Rftn1, Fam107b, Txndc12, Tm7sf3, Ermp1, Pon2, Abl1, Iffo1, Idi1, Plekha2, Ctso, Sept2, Ankrd28, Sytl5, Pde4b, Cdh20, Arrdc3, Rassf2, Mfsd13a, Snx15, Rhob, Slc35b2, Ddit4, Tmem144, Bin1, S100a6, Tirap, Slc25a35, Cpd, Kmt5a, Gpm6b, Rbks, Xaf1, Asrgl1, Shroom1, Sccpdh, Plk3, Cln8, Elovl6, Ap5z1, Zfp276, Carhsp1, Slc22a23, Prox1, Endod1, Dhcr7, Usp6nl, Lzts2, Rab33a, Casq1, Csf1, Rcbtb1, Arhgef37, Nudt4, Necap2, Acsl1, Omg, Fbxo7, Tmem163, Cyp4f13, P3h4, Ipo13, Rhbdl2, Scd1, Ephb1, Dnajb2, Col9a1, Mid1ip1, H2afj, Capn5, Pim3, Tmem268, Atg4c, Epb41l2, Vmp1, Txndc16, Cadm4, Nkain1, Ilvbl, Mfsd14a, Shcbp1l, Rras2, Igsf11, Afap1l1, Msmo1, Tspan9, Wrap53, Aplp1, Leprot, Fam63a, Pik3c2b, Specc1, Dnajc24, Aspscr1, Rhoc, Zfp536, Hspa2, Glul, Aacs, Ubl7, Chmp1b, Fmnl2, Syde2, Ptpdc1, Frmd4b, Gstk1, Chadl, Ttll7, Spns2, Dnm2, Mylpf, Fut10, Lrrc8b, Cercam, Elovl5, Mitf, Stmn4, Dock1, Fam222a, Mad1l1, Tmc7, Lmf1, Bricd5, Npat, Il18, Fam122b, Mon2, Rnpepl1, Ahrr, Fzd5, Hebp1, Inf2, Gca, Cdc37l1, Mtmr2, Sema5a, Rasgrp3, Grhpr, Mta3, Sall1, Hist1h2bn, Osbpl7, Enoph1, Cdr2l, Pdgfc, Ptp4a1, Ubl3, Natd1, Rftn2, Map3k11, Cep97, Gnptab, Fpgt, Bace1, Cyb5r2, Nkx2-2, Ralgds, Prkd3, Nectin1, Trim36, Car13, Wnk1, Hr, Urm1, Sept7, St3gal4, Ctnnal1, Slco3a1, Hist1h2bf, Asap3, Hist1h2bm, Fdps, Mospd2, Arap2, Apln, Mvp, Edil3, Mvd, Rap2a, Dennd5a, Kif13a, Adamts2, Zfyve21, 4930402H24Rik, Acy1, Fntb, Slc9a3r2, Fgd3, Lamp2, Ptn, Slc25a13, Stard13, Erf, Cd3d, Rph3al, Sohlh2, Prickle1, Klf13, Zfp14, Usp30, Acsl3, Papss1, Larp6, Rhoa, Snap23, Zcchc24, Tmem117, Rnls, Fkbp9, Man1a, Sft2d2 |
| Radial glia-like cell | Olfr357, Olfr453, Fabp7, 1700029H14Rik, Sh2d4a, Ghrhr, Shisa3, Fgfbp3, Ptx3, Cga, Veph1, Tnc, Dytn, Xcr1, Wbscr28, Has2, Fshb, Lhcgr, Lipg, 1700012P22Rik, Fcrl5, Slc17a4, Col2a1, Tnnt3, Reg3b, Cybrd1, Wfdc5, Aldh1l1, Ccnb3, Mdfi, Fgf15, Cyp26b1, Sulf1, Celsr1, Pax3, Foxd4, Gprc6a, Glipr1l2, P4ha3, Nkx2-9, Depdc1a, Ska3, Hes5, Lrp2, Kcne1l, Hist1h3g, Troap, Cdc25c, Pipox, Ntn1, Myoz3, Tdgf1, Cfap100, Mgst1, Ube2c, Prss35, Fzd9, Prc1, Krt81, Spc25, Nek2, Ska1, Ckap2, Ednrb, Hmmr, Cdca5, Tk1, Cdca3, Gm17455, Fam64a, Kif4, Cdc20, Dlgap5, Itga2, Erbb2, Plk1, Nusap1, Lrr1, Kif18b, Bub1, Aspm, Gcm1, Tspan18, Dnajb13, Spdl1, Sgol1, Cep55, Adgrv1, Col22a1, Kif23, Spc24, Kif11, Rdh8, Cenpf, P2rx6, Cdk1, Itgb5, Dscc1, Pclaf, Arhgef39, Birc5, A2m, Tpx2, Pbk, Cldn19, Frem2, Tlr9, Oip5, Kif18a, Dpep3, Cdca8, Phgdh, Iqgap3, Top2a, Hmgb4, Nkx2-4, 2810459M11Rik, Aurkb, Vim, Lxn, Nrarp, Ncapg, Trpv5, Alpl, Ndc80, Ribc2, Pif1, Cks2, Dio1, Melk, Knstrn, Ccdc190, Fgr, Reg4, G6pc2, Cd36, Kif20a, Kif15, Gdpd2, Fzd2, Vcam1, Ckap2l, Kif2c, Rnf148, Tead3, Nuf2, Hmgb2, Brip1, Mroh9, Foxb1, Ccdc60, Cenpn, Mki67, Mis18bp1, Mastl, Notch3, Shmt1, Trim31, Ccna2, Fndc11, Capsl, Ect2, Cenpe, Plekhg2, Lrig1, Spon1, Heph, Tacc3, Hist1h2ae, Gins2, Ttk, Vit, Rfx4, Ncaph, Tead2, Chtf18, Hist1h3h, Upp1, Akap14, Ajuba, Stil, Ccnb2, Gpx8, Fmo1, Fgfr3, Efcab11, Dnaaf3, Hist1h3c, Olfr557, Cfap126, Sox21, Cenph, Sall3, Lfng, Tgif2, Cdkn3, Glb1l2, Esco2, Npl, Gcnt3, Cenpa, Kif20b, Got1l1, Ccdc3, Treh, Mcm10, Gsg2, Cdca2, Wnt7b, Hist1h3d, Sema3b, Gtse1, Uhrf1, Brca1, Eya4, Dnajb7, Mdk, Isl2, Smo, Pdlim4, Efna4, Fam83d, Mettl24, Bub1b, Kcnn4, Foxn1, Shcbp1, Tcf7l1, Timp4, Pgpep1, Pax4, Plat, Pxdc1, Acss1, Slc2a10, Cenpm, Pole2, A1bg, Myh8, Srpx, Eme1, Pdpn, Gli1, Ptgr1, E2f8, Shh, Exo1, Sfrp2, Foxm1, Ptch1, Chmp4c, Cdc45, Ttc23, Mxd3, Spef2, Gm1661, Mfap2, Sox3, Pawr, Smc4, Cenpi, Zwilch, Ptprh, Hist1h2ag, 4930505A04Rik, Fanca, Hist1h3b, Notch1, Cngb3, Zar1l, Clhc1, Prelid2, Spata17, Rrm2, Neil3, Cdk6, Hist1h2ab, Bora, Hist1h3i, Pnliprp1, Sfrp1, Mtfr2, Metrn, Mlph, Tex33, Tusc5, Gmnc, Slc15a2, Ednra, Cks1b, Spag8, Fam181b, Zp1, Traf1, Depdc1b, Atp7b, Fermt1, Rnf180, Evx1, Crb2, Id1, Fbxo5, Cenpq, Morn3, Plce1, Abcc12, Smoc1, Dbi, Eppk1, Irx2, Amelx, Clec3b, Mefv, Arhgap11a, Cxcr4, Cyp39a1, Slc25a18, Cdk2, Cplx4, Dppa4, Prom1, Hist2h3c2, Bdh2, Tspan11, Mad2l1, Kif14, Hpse, Chst3, Wdr38, Asf1b, Serpina11, Corin, Mns1, Hist1h2ad, Tnfaip8, Gmnn, Rp1, Sall1, Adgrg6, Fam83b, Scnn1g, Adam30, Fam221a, 1700028P14Rik, Sox9, Espl1, Bnc1, Hopx, Rbl1, Mcm5, Ttpa, Tmprss5, Dnph1, Pnma5, Uts2r, Neurog1, Hist1h2ai, Ube2t, Smc2, Olfr658, Muc5ac, Vgll3, Chek1, Emp1, Prr11, Cdca7l, Rgma, Slc43a1, Ccdc187, Spag5, Hist1h2af, Fignl1, Gins1, Kif22, Wls, Fads2, E2f5, Mest, Kcnk5, Kcnq4, 4933413G19Rik, Klb, Otx2, Nkain4, Nde1, Cenpw, Mcm2, Slc45a2, Mlf1, Cenpp, Slc17a2, Slc2a4, Dpep1, Actbl2, Galnt10, Mcm3, Ccnf, Helt, Kpna2, Megf10, BC055324, Kcnv2, Card11, Rrm1, Mylk4, Tbc1d4, Gas2l2, Slc1a3, Gas2l3, Krt40, Lmnb1, Cdc6, St5, Enkur, Mcm6, Incenp, Hyal1, Pygl, Cbx2, Slc9a3r1, Slc44a4, Nat2, Igdcc3, Sema5b, Olfr57, Olfr64, Ncapd2, Aplf, Ptprz1, Slc17a3, Aurka, Cpxm1, Acsm2, Id3, Sox2, Rdm1, Nsl1, Fam181a, Hist2h3b, Kyat3, H2afv, Col5a2, C330027C09Rik, Cth, Hey2, Cnn3, Serpine2, Racgap1, Adgrg3, 1700113H08Rik, Zbtb42, Pigr, Epha3, Yap1, Tcf19, Tmem139, Itgb8, Ccdc114, Hvcn1, Cdc14a, Fzd7, Prdm6, Adtrp, Tph1, Gpsm2, Reep4, Casq1, Cenpk, Folr1, Mtbp, Cdo1, Msx2, Pard6b, Vsx2, Bard1, Lix1l, Tuba1c, Myom2, Slc5a9, Hist1h3a, Haus5, Ccr8, Ccdc18, Dsn1, Tyms, Chsy1, Lexm, Fga, Suclg2, Cchcr1, Mmp14, Acadl, Grk1, Adhfe1, Igf2bp2, Polq, E2f2, Tmem184a, Hes1, Hus1, Slc6a11, 4930427A07Rik, Nuak2, Wnt7a, Kif12, Nedd9, Odf3l1, Mfge8, Cdca7, Pkn3, Acaa2, Hist1h4d, Znrf4, Ddias, Fancb, Kdelr3, Aif1l, Heatr9, Hells, Egfr, Ddx11, Ankle1, Opn1sw, Cand2, Cd1d1, Kirrel2, Slc30a8, Trim60, Trip13, Epb42, Cpt1a, Afap1l2, Mybl2, Cr2, C1qtnf6, Kntc1, Vangl2, Slc22a7, Mcm9, Grxcr2, Etv4, Pkmyt1, Tom1l1, Map7d3, Abcg8, Six5, 4932438H23Rik, Arsi, Acss3, Chek2, Hist1h2bn, Nr4a3, Ccdc96, Rab38, Matn3, Clic1, Ccdc34, Tjp3, Jam2, Serpinh1, E2f7, Kcnj15, Myom3, Efemp2, Gdf5, Fkbp9, Hist1h3f, Lhfp, Car3, Igfbp2, Cfap43, Bcl2l12, Klhl25, Pifo, Mcm7, Kif7, Stbd1, Dtl, Ppp1r3c, Rag1, Treml2, Casp6, Psmc3ip, Cdk4, Psrc1, Rab13, Cldn1, Fam167a, Slc30a2, Galnt4, Iqcg, Arhgef26, Lsm2, Dbf4, Loxl3, Ticrr, Mia, Hs3st3b1, Epgn, Plk4, Calml4, Tnni3, Cenpu, Dhfr, Fbxl7, Pold1, Ttyh1, Frem1, Ecm2, Lrat, Igf2bp1, Stpg2, Parpbp, Cep128, Msi1, Rad51ap1, Bcl2, Irx3, Wdr62, Rhpn1, Sncaip, Hist1h2an, Egln3, Pttg1, Armc3, Rhbdf1, Pard3b, Irx1, Timeless, Hist1h3e, Trp53i13, Gsdma, Cfap77, Slc25a13, Emp2, Chaf1a, Ttll9, Cenpl, Serinc2, Nedd1, Ptbp1, Ccr2, Dynlrb2, Rfc4, Kcnj16, Ubxn10, Rcc1, Palld, Pxmp2, Agpat2, Phactr4, Plod1, Fgfrl1, Ccdc36, Apoh, Psapl1, Trip6, 2610020H08Rik, Lpar4, Spata13, Fst, Col23a1, Hapln3, Ptpn13, Fgf6, Spin4, Mre11a, Clspn, Srebf1, Caskin2, Cfap53, Nqo1, Foxj1, Ezh2, Tmprss4, Col27a1, Vps37b, Rad54l, Sapcd2, Rfc5, Gins3, Ppic, Pi15, Rassf4, 2410004P03Rik, Ccdc73, Fzd10, Ankef1, Cfap44, Sfrp5, Plag1, Gm973, Fmo4, Trpm6, Rnls, Hist1h4i, Vwa7, Itprip, Mamdc2, Skp2, St14, Chaf1b, Kif6, Ano1, Ccnd2, Cep152, Pbxip1, Magt1, Zfp217, Sulf2, Krt28, Plekhf2, Nr2f1, Xirp1, Tril, Hist2h2ab, Fam69c, Recql4, Fstl1, Lrrc17, Hist1h2bb, Setdb2, Tmem82, Jag1, Lrig3, Adgrg1, B3gnt5, Poc1a |

**Supplementary Table 2.** Results of the analyses of the associations between cell type-specific polygenic risk scores (radial glia, PRS^Rad^; oligodendrocyte precursor cells, PRS^OPC^; and mature oligodendrocytes, PRS^Oli^) and volumetric changes between baseline (V1) and 3 months (V3) in the left CA4/dentate gyrus (CA4/DG) and right CA4/DG in the 3 subgroups included in this study. All results are corrected for sex, age, height, handedness, and 2 ancestry principal components. Adjusted p values are shown. HC: healthy controls; SCZ: schizophrenia; PRS: polygenic risk score.

|  |  |  | **CA4/DG LEFT** | | | **CA4/DG RIGHT** | | |
| --- | --- | --- | --- | --- | --- | --- | --- | --- |
| **Intervention group** | **Cell type-specific PRS** | **Threshold** | **Effect PRS** | **Adjusted P value PRS** | **R^2^ change** | **Effect PRS** | **Adjusted P value PRS** | **R^2^ change** |
| HC_endur_training | PRS^Rad^ | 5x10^-8^ | -0.002520344 | 0.843137255 | -0.016676539 | -0.00179574 | 0.541176471 | 0.089529145 |
|  |  | 1x10^-6^ | -0.003003715 | 0.432835821 | -0.00136581 | -0.001940611 | 0.473684211 | 0.097521334 |
|  |  | 1x10^-4^ | -0.001453591 | 0.573333333 | -0.024564685 | -0.00142682 | 0.352941176 | 0.098456809 |
|  |  | 1x10^-3^ | -0.00118861 | 0.619047619 | -0.017873961 | 6.68E-05 | 1 | 0.062840606 |
|  |  | 0.01 | -0.001187248 | 0.573333333 | -0.021828935 | -0.00073201 | 0.633333333 | 0.078873502 |
|  |  | 0.05 | -0.001373161 | 0.444444444 | 0.002619765 | -6.01E-05 | 1 | 0.062856492 |
|  |  | 0.1 | -0.002146388 | 0.222857143 | 0.088942199 | -0.000737087 | 0.642857143 | 0.093796666 |
|  |  | 0.2 | -0.002043447 | 0.125177809 | 0.12313129 | -0.000183038 | 0.784313725 | 0.06534593 |
|  |  | 0.5 | -0.001516808 | 0.193317422 | 0.063459221 | -8.67E-05 | 1 | 0.063376818 |
|  |  | 1 | -0.001560054 | 0.291497976 | 0.085370205 | -2.22E-05 | 1 | 0.062733214 |
|  | PRS^OPC^ | 5x10^-8^ | 0.000265451 | 1 | -0.043056679 | -0.008059407 | 0.170040486 | 0.242256851 |
|  |  | 1x10^-6^ | 0.006450824 | 0.308370044 | 0.05413165 | -0.00170445 | 0.637931034 | 0.076247126 |
|  |  | 1x10^-4^ | 0.005946147 | 0.177730193 | 0.075589817 | 0.002160325 | 0.527472527 | 0.093988306 |
|  |  | 1x10^-3^ | 2.03E-05 | 1 | -0.043150427 | 0.001442476 | 0.505050505 | 0.100919146 |
|  |  | 0.01 | 0.000894819 | 0.921568627 | -0.032049078 | 0.00124969 | 0.745098039 | 0.105944884 |
|  |  | 0.05 | 0.000883319 | 0.901960784 | -0.029863029 | 0.001424837 | 0.510204082 | 0.131756771 |
|  |  | 0.1 | 0.00024876 | 0.941176471 | -0.041127852 | 0.000731055 | 0.52173913 | 0.097636948 |
|  |  | 0.2 | -0.000778463 | 0.882352941 | -0.020045916 | 0.000680793 | 0.379518072 | 0.097982146 |
|  |  | 0.5 | -0.001313611 | 0.413793103 | 0.006708199 | 0.000525157 | 0.705882353 | 0.0785989 |
|  |  | 1 | -0.001235972 | 0.583333333 | 0.000554829 | 0.000766692 | 0.705882353 | 0.096275118 |
|  | PRS^Oli^ | 5x10^-8^ | 0.004591499 | 0.62295082 | 0.006302163 | -0.002895732 | 0.420289855 | 0.101972331 |
|  |  | 1x10^-6^ | 0.003032421 | 0.633333333 | -0.017519718 | -0.00205899 | 0.862745098 | 0.086286948 |
|  |  | 1x10^-4^ | 0.000997562 | 1 | -0.039603418 | -0.002206007 | 0.555555556 | 0.097364765 |
|  |  | 1x10^-3^ | -0.000306183 | 0.980392157 | -0.04262641 | -0.000407094 | 0.764705882 | 0.064544699 |
|  |  | 0.01 | -0.001695736 | 0.745098039 | -0.013652415 | 0.000861154 | 0.843137255 | 0.07787813 |
|  |  | 0.05 | -4.75E-05 | 1 | -0.043122976 | 0.001932683 | 0.220338983 | 0.166092767 |
|  |  | 0.1 | 0.000429011 | 0.980392157 | -0.039961274 | 0.001243215 | 0.382716049 | 0.11623754 |
|  |  | 0.2 | 0.000317416 | 0.941176471 | -0.040762421 | 0.001164061 | 0.352941176 | 0.126932341 |
|  |  | 0.5 | 0.000333285 | 0.882352941 | -0.038690999 | 0.000494173 | 0.725490196 | 0.0822803 |
|  |  | 1 | 0.000493028 | 0.637931034 | -0.033066434 | 0.000657105 | 0.571428571 | 0.09847319 |
| SCZ_endur_training | PRS^Rad^ | 5x10^-8^ | -0.011588972 | 0.0158 | 0.448916705 | -0.006010481 | 0.181619256 | 0.086829326 |
|  |  | 1x10^-6^ | -0.010121132 | 0.02776204 | 0.29745692 | -0.005676904 | 0.090196078 | 0.063434444 |
|  |  | 1x10^-4^ | -0.007499191 | 0.034263511 | 0.288162811 | -0.003713095 | 0.189814815 | 0.032538816 |
|  |  | 1x10^-3^ | -0.004769276 | 0.039884868 | 0.281723227 | -0.003360415 | 0.073459716 | 0.130084293 |
|  |  | 0.01 | -0.004245907 | 0.035043353 | 0.307632253 | -0.004196928 | 0.0064 | 0.345946446 |
|  |  | 0.05 | -0.003105573 | 0.0178 | 0.392523249 | -0.002798372 | 0.005 | 0.347654055 |
|  |  | 0.1 | -0.00255495 | 0.04033264 | 0.310383079 | -0.002150472 | 0.034421576 | 0.234747977 |
|  |  | 0.2 | -0.001864506 | 0.080843585 | 0.221075327 | -0.001533374 | 0.059153176 | 0.158650237 |
|  |  | 0.5 | -0.001779062 | 0.060509554 | 0.224333604 | -0.00157729 | 0.048533873 | 0.197708089 |
|  |  | 1 | -0.001768861 | 0.055232558 | 0.24554745 | -0.001502923 | 0.040591966 | 0.192050385 |
|  | PRS^OPC^ | 5x10^-8^ | -0.014014802 | 0.040099215 | 0.275228238 | -0.00898758 | 0.11573472 | 0.093695815 |
|  |  | 1x10^-6^ | -0.010863074 | 0.096602972 | 0.212133418 | -0.007863409 | 0.161290323 | 0.103229881 |
|  |  | 1x10^-4^ | -0.007759265 | 0.034421576 | 0.419779186 | -0.002233411 | 0.372781065 | -0.021318742 |
|  |  | 1x10^-3^ | -0.00320797 | 0.062416999 | 0.16263249 | -0.000166193 | 1 | -0.065119527 |
|  |  | 0.01 | -0.003803388 | 0.0092 | 0.444014802 | -0.001638405 | 0.312217195 | 0.038539908 |
|  |  | 0.05 | -0.002344043 | 0.038294512 | 0.271816199 | -0.001396658 | 0.232628399 | 0.07062538 |
|  |  | 0.1 | -0.001985938 | 0.06871345 | 0.265919559 | -0.00124704 | 0.136363636 | 0.083322267 |
|  |  | 0.2 | -0.001825422 | 0.067238913 | 0.254591156 | -0.001263872 | 0.091733871 | 0.10989108 |
|  |  | 0.5 | -0.001635015 | 0.070938215 | 0.249264685 | -0.001029649 | 0.242811502 | 0.077353224 |
|  |  | 1 | -0.001680426 | 0.038188976 | 0.282151357 | -0.001059402 | 0.093717817 | 0.091186521 |
|  | PRS^Oli^ | 5x10^-8^ | -0.003781674 | 0.606060606 | -0.005397916 | 0.00077775 | 0.980392157 | -0.061717085 |
|  |  | 1x10^-6^ | -0.004738952 | 0.174369748 | 0.039000562 | 0.000444381 | 0.960784314 | -0.064585505 |
|  |  | 1x10^-4^ | -0.003664457 | 0.27443609 | 0.04124728 | 0.001037032 | 0.745098039 | -0.054162043 |
|  |  | 1x10^-3^ | -0.000614172 | 0.882352941 | -0.093572928 | 0.003157333 | 0.089494163 | 0.104872675 |
|  |  | 0.01 | -0.000740943 | 0.921568627 | -0.07765085 | 0.002115371 | 0.131147541 | 0.121047138 |
|  |  | 0.05 | -0.000652196 | 0.591549296 | -0.064551232 | 0.001180939 | 0.212938005 | 0.053756702 |
|  |  | 0.1 | -0.000298223 | 0.725490196 | -0.093296615 | 0.001764961 | 0.0363432 | 0.170425384 |
|  |  | 0.2 | -0.000166329 | 0.921568627 | -0.097020369 | 0.00139748 | 0.111809045 | 0.138439931 |
|  |  | 0.5 | -0.000433169 | 0.619047619 | -0.076748659 | 0.000978223 | 0.211081794 | 0.055849309 |
|  |  | 1 | -0.000587007 | 0.473684211 | -0.05858351 | 0.000949188 | 0.666666667 | 0.045674547 |
| SCZ_table_soccer | PRS^Rad^ | 5x10^-8^ | -0.00384327 | 0.637931034 | -0.051151101 | 0.001931921 | 0.564102564 | 0.257667865 |
|  |  | 1x10^-6^ | -0.003128719 | 0.423357664 | -0.069502258 | 0.002275574 | 0.42962963 | 0.292747797 |
|  |  | 1x10^-4^ | -0.003579944 | 0.555555556 | -0.011867976 | 7.96E-05 | 1 | 0.205390409 |
|  |  | 1x10^-3^ | -0.004614986 | 0.547619048 | 0.058485307 | 0.000441004 | 0.843137255 | 0.209432168 |
|  |  | 0.01 | -0.00855361 | 0.100775194 | 0.353274498 | 0.000202558 | 1 | 0.205879476 |
|  |  | 0.05 | -0.002153096 | 0.666666667 | -0.110070164 | 0.002572806 | 0.19025522 | 0.308695025 |
|  |  | 0.1 | 0.00045437 | 0.960784314 | -0.139445738 | 0.001012453 | 0.510416667 | 0.230739012 |
|  |  | 0.2 | 0.001561111 | 0.921568627 | -0.109178443 | 0.000614132 | 0.745098039 | 0.216772238 |
|  |  | 0.5 | 0.002161173 | 0.660377358 | -0.097262475 | 0.001761592 | 0.372093023 | 0.272880995 |
|  |  | 1 | 0.002076459 | 0.862745098 | -0.104959618 | 0.001464551 | 0.473214286 | 0.247111683 |
|  | PRS^OPC^ | 5x10^-8^ | -0.01032017 | 0.347368421 | 0.138623502 | 6.76E-05 | 1 | 0.205270958 |
|  |  | 1x10^-6^ | -0.007320209 | 0.5 | 0.101386596 | 0.000278306 | 1 | 0.206048548 |
|  |  | 1x10^-4^ | -0.009243816 | 0.075671277 | 0.346749059 | 0.000269435 | 0.941176471 | 0.206194339 |
|  |  | 1x10^-3^ | -0.009805955 | 0.036868111 | 0.564859046 | -0.000402635 | 0.862745098 | 0.207973154 |
|  |  | 0.01 | -0.004572638 | 0.070517629 | 0.354961212 | 0.000336767 | 0.882352941 | 0.211416625 |
|  |  | 0.05 | -0.002716027 | 0.335 | 0.062882511 | 0.001032298 | 0.345549738 | 0.272963992 |
|  |  | 0.1 | -0.001158584 | 0.666666667 | -0.112849729 | 0.002046511 | 0.095287958 | 0.41142898 |
|  |  | 0.2 | 7.11E-05 | 1 | -0.141595031 | 0.00249913 | 0.072373541 | 0.463879451 |
|  |  | 0.5 | 0.00054406 | 0.784313725 | -0.133819601 | 0.002295841 | 0.085740913 | 0.526263783 |
|  |  | 1 | 0.000843882 | 0.882352941 | -0.122904317 | 0.002325001 | 0.044260028 | 0.531957668 |
|  | PRS^Oli^ | 5x10^-8^ | 0.001221522 | 0.921568627 | -0.137519131 | 0.005173646 | 0.150442478 | 0.376556826 |
|  |  | 1x10^-6^ | 0.001832054 | 0.862745098 | -0.12963849 | 0.005288913 | 0.233333333 | 0.435340143 |
|  |  | 1x10^-4^ | -0.002287978 | 0.68627451 | -0.094319888 | 0.0012612 | 0.5625 | 0.238225299 |
|  |  | 1x10^-3^ | -0.003289471 | 0.330049261 | 0.00191843 | 0.000990635 | 0.666666667 | 0.235089172 |
|  |  | 0.01 | -0.002113791 | 0.5 | -0.049330132 | 0.001020746 | 0.361111111 | 0.254596742 |
|  |  | 0.05 | -0.002639994 | 0.392405063 | 0.075009162 | 0.000231516 | 0.784313725 | 0.209062355 |
|  |  | 0.1 | -0.002312437 | 0.440944882 | 0.061162827 | -7.16E-05 | 1 | 0.205689106 |
|  |  | 0.2 | -0.001905151 | 0.490384615 | 0.017880571 | -0.000166178 | 0.823529412 | 0.208025493 |
|  |  | 0.5 | -0.001394697 | 0.533333333 | -0.046216692 | 0.000221515 | 0.980392157 | 0.210762287 |
|  |  | 1 | -0.001426362 | 0.784313725 | -0.026074223 | 0.000155366 | 0.921568627 | 0.208386765 |
